# Toxic compound, morphology, biology, and phenology of the calcinogenic plant *Nierembergia rivularis* Miers (Solanaceae)

**DOI:** 10.1016/j.toxcx.2026.100259

**Published:** 2026-06-24

**Authors:** Marcela Preliasco, Ary Mailhos, Facundo Ibáñez, Alejo Menchaca, Aldana Balserini, Ana Celina González, Franklin Riet-Correa, Rodolfo Rivero

**Affiliations:** a División de Laboratorios Veterinarios, Ministerio de Ganadería, Agricultura y Pesca, Ruta 8 Km 17.500, Montevideo, 12100, Uruguay; b Departamento de Biología Vegetal, Facultad de Agronomía, Universidad de La República, Garzón 780, Montevideo, 12900, Uruguay; c Plataforma de Agroalimentos, Instituto Nacional de Investigación Agropecuaria, Estación Experimental Las Brujas, Ruta 48 Km 10, Canelones, 90100, Uruguay; d Plataforma de Investigación en Salud Animal, Instituto Nacional de Investigación Agropecuaria, Estación Experimental La Estanzuela, Ruta 50 Km 11, Colonia, 70000, Uruguay; e División de Sanidad Animal, Ministerio de Ganadería Agricultura y Pesca, Sarandí 403, Paso de Los Toros, Tacuarembó, 45100, Uruguay; f Programa de Pós-graduação em Ciências Animais nos Trópicos, Universidade Federal da Bahia, Av. Adhemar de Barros 500, Ondina, Salvador, 40170-110, Brazil

**Keywords:** Plant anatomy, Plant phenology, Uruguayan flora, Poisonous plants, Enzootic calcinosis, Vitamin D poisoning

## Abstract

Enzootic calcinosis caused by *Nierembergia* spp*.* (Solanaceae) poisoning causes important economic losses for sheep and cattle production in South America. This study aims to provide a comprehensive description of *N. rivularis* Miers, focusing on its morphological characteristics, biomass distribution, dry matter content, phenological cycle, floral biology, environmental growth preferences, and the presence of potentially toxic compounds. The results revealed that *N. rivularis* is a perennial, small-sized plant whose stems are modified into underground rhizomes. These subterranean structures feature nodes from which adventitious roots emerge, functioning as the primary mechanism for clonal asexual reproduction. Its white, pentamerous flowers display capitate trichomes in the corolla throat and a long, firm corolla tube that positions the corolla semi-parallel to the soil. Two insect species were observed interacting with flowers of *N. rivularis*, both during the spring season: *Astylus quadrilineatus* (Melyridae) and a small bee from the family Apidae, subfamily Apinae, tribe Tapinotaspidini. The plant exhibited a belowground biomass (rhizomes and roots) up to 15 times greater (dry weight based) than its aerial structures (leaves and flowers). The greater presence of *N. rivularis* in the fields was associated with high soil humidity, direct sunlight exposure, mixed soil texture, and low competition from rhizomatous species for underground space. Hydroxylated vitamin D derivatives were detected by HPLC in all structures of *N. rivularis*, suggesting this species' capacity to accumulate calcinogenic substances. Knowledge about the toxic compounds, morphology, biology, and phenology of *N. rivularis* will contribute towards the prevention and control of poisoning by this plant.

## Highlights

• *Nierembergia rivularis* is distributed throughout Uruguay, Argentina, Bolivia, and Brazil.
• Collective outbreaks of enzootic calcinosis have been reported in sheep and cattle in Uruguay.
• On average, this rhizomatous plant allocates 81.3% of its total dry biomass to underground structures.
• It is a perennial plant that grows mainly in high-humidity, fine-to-coarse textured soils.
• *Nierembergia rivularis* accumulates hydroxylated derivatives of vitamin D.

## 1 Introduction

Enzootic calcinosis refers to the clinical and pathological condition observed in animals consuming calcinogenic plants. These plants contain active compounds which, through a complex pathogenesis that involves genomic and non-genomic effects, leads to the mineralization of soft tissues, particularly the tunica intima and media of elastic arteries (Worker and Carrillo, 1967; Tokarnia et al., 2000; Mello, 2003; Rissi et al., 2009; Machado et al., 2020a, 2022). This condition results in a chronic emaciation syndrome, which often culminates in death due to cardiac and respiratory failure (Worker and Carrillo, 1967; Tokarnia et al., 2000; Mello, 2003; Machado et al., 2020a, 2020b). Several plants, including *Solanum glaucophyllum* Desf., *Nierembergia riograndensis* Hunz. & A.A. Cocucci (named as *Nierembergia veitchii* Hook. in reports of enzootic calcinosis outbreaks in Brazil), *Nierembergia rivularis* Miers., *Cestrum diurnum* L., *Trisetum flavescens* (L.) P. Beauv., *Solanum torvum* Sw., *Solanum esuriale* Lindl., *Solanum stuckertii* Bitter, and *Stenotaphrum secundatum* (Walter) Kuntze, have been identified as calcinogenic in different regions of the world (Worker and Carrillo, 1967; Morris et al., 1979; Riet-Correa et al., 1987; Arnold and Fincham, 1997; Durand et al., 1999; Braun et al., 2000; Mello, 2003; Vignoli-Silva and Mentz, 2006; Iglesias et al., 2008; García y Santos et al., 2012; Machado et al., 2020a; Rossanigo et al., 2020). Enzootic calcinosis leads to significant economic losses in agricultural systems, manifesting as weight loss, reduced feed conversion rates, decreased carcass value, lower reproductive rates and animal mortality (Tokarnia et al., 2000; Mello, 2003; Machado et al., 2020a; Schild et al., 2021).

**Fig. 1:**
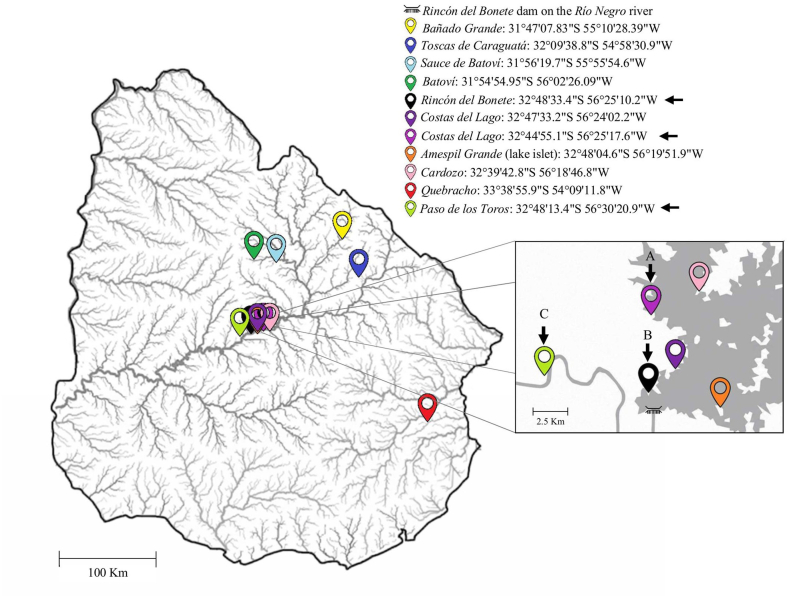
Map of Uruguay showing the locations of the 11 farms where a total of 15 outbreaks of enzootic calcinosis attributed to *Nierembergia rivularis* poisoning were reported over a 15-year period. The three farms (A, B, and C) where the experiment was conducted are marked with arrows. A schematic representation of the main hydrography is shown for descriptive purposes only.

*Nierembergia rivularis* (Solanaceae) has been identified as an etiological agent of enzootic calcinosis in Uruguay, with its calcinogenic properties experimentally confirmed in both sheep (García y Santos et al., 2012) and cattle (Schild et al., 2021). A total of 15 outbreaks of enzootic calcinosis caused by *N. rivularis* poisoning were previously reported in both scientific literature (García y Santos et al., 2012; Machado et al., 2020b; Schild et al., 2021) and records from the Diagnostic Analysis and Records Unit (UNIRADD) of the Ministry of Livestock, Agriculture and Fisheries (MGAP) of Uruguay, affecting 11 farms from 2006 to 2021 (Fig. 1). The specific epithet *rivularis* derives from the Latin *rivulus*, meaning ‘small stream,' and indicates that the species typically grows near watercourses or in moist habitats. This creeping plant is distributed throughout the hydrographic system of the *Río Paraná* and *Río de la Plata* rivers, covering regions of Argentina, Bolivia, Brazil, and Uruguay (Cocucci and Hunziker, 1995; Vignoli-Silva and Mentz, 2020). In Uruguay particularly, it is distributed throughout the southwest, center, and southeast of the country, associated with major watercourses such as the *Río Uruguay*, *Río Negro*, *Río de la Plata*, and *Cebollatí* rivers, as well as the *Laguna Merín* lagoon (Marchesi et al., 2026).

The development of effective control strategies for *N. rivularis* has been challenging due to the limited information available about its morphological and physiological characteristics. For instance, there are conflicting reports regarding its phenological cycle. Some authors in Uruguay describe it as an annual species present in the field during spring and summer (García y Santos et al., 2012), coinciding with outbreaks of enzootic calcinosis, a highly seasonal disease. In contrast, other authors in Argentina (Millán, 1941; Zuloaga et al., 2025) and Brazil (Vignoli-Silva and Mentz, 2020) classify its cycle as perennial.

Within the framework of neotropical oil-flower evolution, typical *Nierembergia* species rely on specialized mutualisms with oil-collecting bees (Cocucci, 1991; Carneiro and Machado, 2023). While recent broad-scale surveys suggested that certain herbaceous lineages within the genus might have functionally lost their oil-secreting capabilities, the micro-morphological conservation and functional state of these secretor structures remain unquantified at regional scales (Tate et al., 2009; Maubecin et al., 2021). Furthermore, due to high morphological homoplasy within this group, *N. rivularis* suffers from persistent taxonomic confusion with close relatives like *N. repens* (Tate et al., 2009). Consequently, there is a lack of quantitative data on its biomass distribution, soil preferences, immediate plant assemblages, and specific toxic compounds. Understanding the basic biology of this species is essential not only to generate foundational data on *N. rivularis*, but also to thoroughly evaluate the environmental impact that its management and control measures could exert on the natural grassland ecosystems it inhabits. Different plant poisonings in nature can be explained by the complex interactions between plants and animals, as well as their nature, growth stages, geographic location, environmental conditions, and livestock species (Rasool et al., 2024). In this context, systematic investigations into year-round morphometrics, biomass allocation, and edaphic drivers are necessary to address these factors under field conditions.

Although it is hypothesized that the toxicity of *N. rivularis* is linked to its ability to accumulate vitamin D derivatives, similar to those found in other calcinogenic plants such as *S. glaucophyllum* (Haussler et al., 1976; Wasserman et al., 1976), this hypothesis remains unconfirmed. The aim of this study was to provide a comprehensive description of the morphology, phenology, and biology of *N. rivularis* and to investigate the presence and accumulation of calcinogenic compounds in this plant.

## 2 Materials and methods

### 2.1 Study of growth habits of *Nierembergia rivularis*

Of the 11 farms where poisoning outbreaks were reported in Uruguay from 2006 to 2021, three were chosen for the experiment (Fig. 1). This choice was based on year-round accessibility, regardless of weather conditions, and the diverse topography of the pastures, which include plains, flat fields, and stony or mountainous terrain. At each location, populations of *N. rivularis* were examined, and plant specimens were collected for taxonomic identification and archived in the Herbarium of the Faculty of Agronomy (MVFA), *Universidad de la República* (UdelaR) in Uruguay under the voucher numbers: Preliasco 1 MVFA, Preliasco 2 MVFA, and Preliasco 3 MVFA. Additionally, detailed observations were made of the growth habits of *N. rivularis*, as well as of the environmental characteristics of the sites where this species grows (topography, flooding potential, proximity to water sources, and exposure to sunlight).

### 2.2 Sites for biological, phenological, and morphometric studies

The phenological and morphometric features of *N. rivularis* were studied at three locations within the Tacuarembó department of Uruguay (designated as sites A, B, and C), where outbreaks of enzootic calcinosis due to *N. rivularis* poisoning were reported. These sites were accessible throughout the year, ensuring consistent sampling conditions. Six sampling sites (A1, A2, B, C1, C2, and C3) were selected based on the heterogeneity of its topography, the presence of *N. rivularis* and associated plant species (Table 1). The presence of *N. rivularis* at each site was classified as low (≤9%), medium (10 to 29%) or high (≥30%) according to the estimated area covered by the aerial part of the plant, assessed at the time of site selection, following the methodology established by Braun-Blanquet (1950). To standardize the assessments, a reference area of 5 m^2^ was evaluated at each sampling site. At Site A, located on the shore of the *Rincón del Bonete* dam reservoir, 120 m from the water, two sampling points were identified. Point A1 features a topographic depression, whereas point A2 features an adjacent elevation. Site B was established in a flooded pasture, 1.5 km away from the shore of the *Rincón del Bonete* dam reservoir, where a 5 m^2^ plot was randomly selected. Location C comprised an inland pasture, 200 m from an inlet of the *Río Negro* river, with three distinct microtopographical conditions, each evaluated within a 5 m^2^ area: dry mounds (C1), intermediate zones between mounds (C2), and low-lying areas adjacent to the mounds (C3). These three microhabitats represented a diverse range of environments where *N. rivularis* populations occur.

**Table 1:** Characteristics of selected sites for studies on *Nierembergia rivularis* in three locations of Uruguay.

ID	Location^a^	Geographical coordinates	Topographic characteristics	Sampling site	Terrain characteristics	Presence of *Nierembergia rivularis*^b^
A	*Costas del Lago*	32°44'55.1"S 56°25'17.6"W	Natural grassland on the shores of hydroelectric dam lake.	A1	Lowland. Wet area.	High
				A2	Land elevation. Dry zone.	Low
B	*Rincón del Bonete*	32°48'33.4"S 56°25'10.2"W	Enhanced natural grassland. Floodable land. No shoreline.	B	Lowland. Wet area.	Medium
C	*Paso de los Toros*	32°48'13.4"S 56°30'20.9"W	Natural grassland. Floodable. Banks of a stream tributary of *Río Negro* river.	C1	Land elevation (mound). Dry zone.	Low
				C2	Intermediate zone.	Low
				C3	Lowland. Wet area.	High

a Location is further illustrated in Figure 1.

b Low (≤9%), medium (10 to 29%) and high (≥30%) presence was defined according to the area covered by the aerial part of the plant (Braun-Blanquet, 1950).

### 2.3 Characterization of plant assemblages in *Nierembergia rivularis* patches

In each sampling site, homogeneous vegetation patches where *Nierembergia rivularis* grows were identified. Because *N. rivularis* is a small herbaceous plant, 0.5 x 0.5 quadrats were randomly established within these patches in order to analyze the assemblage of plants growing in immediate proximity of *N. rivularis*. Vegetation surveys were carried out in the spring of 2022. All vascular plant species within the quadrats were identified, and specimens requiring microscopic analysis were collected for identification at the Botany Laboratory of the Faculty of Agronomy (UdelaR). Each site included two to three replicates. Species were assigned a visually estimated aerial cover value (Braun-Blanquet, 1950), along with estimations of dry matter and bare soil cover (Dengler et al., 2008). Only species with a cover of 5% or more in at least one of the replicates were considered in the final analysis.

Observations on the aerial (leaves and flowers) and belowground (rhizomes and roots) biomass of *N. rivularis* were made in relation to the degree of competition across the different sampling sites. The stems of this species consist of underground rhizomes with nodal growth points that direct foliar and flower development upward and root systems downward. Accordingly, for the purpose of this study, these structures were categorized as belowground biomass.

### 2.4 Physicochemical soil properties at *Nierembergia rivularis* sampling sites

Soil samples were collected from sampling sites A1, A2, B, and C3. From each location, twenty random sub-samples (2.5 cm diameter x 30 cm depth) were obtained using a soil auger and then combined to form a single final sample for analysis. Sites C1 and C2 were excluded due to their rocky and compacted soil texture, which hindered the collection of representative samples.

These samples were placed under refrigeration (4°C) in portable coolers within 1 h after field collection, and were subsequently transported to the Soil, Plant, and Water Laboratory of the National Agricultural Research Institute (INIA). Once there, the soils were analyzed for pH (Beretta et al., 2014a) and texture (Beretta et al., 2014b). Then the material was dried at 40°C, ground, and sieved to 2 mm for the analysis of nitrogen (combustion at 900°C followed by N_2_ detection via thermal conductivity), carbon (Wright and Bailey, 2001), phosphorus (Bray and Kurtz, 1945), macronutrients (calcium, magnesium, potassium, and sodium) (Jackson, 1964), micronutrients (copper, iron, manganese, and zinc) (Raij et al., 2001), cation exchange capacity, base saturation percentage, and titratable acidity at pH 7 (Beretta et al., 2017).

### 2.5 Phenological cycle of *Nierembergia rivularis*

Monthly visits to the sampling sites were conducted over the course of one year (March 2021 – February 2022) to document the phenological stages of *N. rivularis*. In addition, to provide a continuous qualitative reference and monitor morphological structures under controlled conditions (free from field confounding factors such as herbivory and interspecific competition), two soil blocks (25 cm^2^ x 20 cm depth) containing *N. rivularis* plants were collected from site A1 and maintained under environmentally controlled conditions at the Toxicology Laboratory of the National Veterinary Laboratories Division (DILAVE) from March 2021 to September 2022. The blocks were placed in plastic containers with drainage, exposed to direct sunlight, and irrigated weekly. Phenological changes and vegetative structures were monitored and descriptively recorded throughout this period as a supportive, non-quantitative validation of the field observations.

### 2.6 Floral dynamics of *Nierembergia rivularis*

#### 2.6.1 Floral cycle

During the monthly field visits, the presence or absence of flowers of *N. rivularis* was recorded, and a 15-min visual observation period was systematically conducted to identify and document insect visitors. At site C, additional observations were conducted to study the floral cycle of *N. rivularis* flowers at different times of the day and night. For that instance, visits were made at 12:00, 17:00, and 22:00 on November 20, 2021, December 15, 2021, and January 22, 2022, to record the opening state of the flowers at each hour, evaluate the presence of nyctinastic movements, and maintain the same 15-min insect observation window within each specific schedule.

#### 2.6.2 Identification of insect species interacting with flowers of *Nierembergia rivularis*

The insects observed interacting with *N. rivularis* flowers during the monthly field visits were collected and sent to the Entomology and Ethology Sections of the Faculty of Sciences (UdelaR) for their taxonomic identification. In this study, ‘interacting' refers to insect species that come into contact with the flowers, whether for feeding, pollination, or other ecological behaviors during each visit.

### 2.7 Morphological studies of *Nierembergia rivularis*

Two soil block samples (25 cm^2^ x 20 cm depth) were taken randomly each month for one year (March 2021 – February 2022) from sampling sites A1 and A2 (n=24). The methodology focused on ensuring the sampling of the entire *N. rivularis* structure, both aboveground (leaves and flowers) and belowground (rhizomes and roots). Soil and debris were removed using stainless steel sieves, plastic containers, and water. From each sample, a total of 10 segments of *N. rivularis* were randomly selected, including all the plant structures (aerial and belowground) (n=240). The morphology of the selected plants was studied and described according to Torres (1966), Cocucci and Hunziker (1995), and Zhang et al. (2018). Morphometric measurements were made using a digital stainless-steel caliper, graduated between 0.01 and 150 mm. At each plant segment, three points were randomly selected to measure the distance between the nodes and the thickness of the rhizomes (720 measurements in total). The aerial parts (leaves and flowers) were then separated from the belowground fractions (rhizomes and roots) using fine-tipped scissors. A total of 30 leaves were randomly selected from each soil block sample (n=720) and the longest length and width of the leaf blades, and the length of the petiole were measured (methodology adapted from Zhang et al., 2018; Wang et al., 2020). The flowers present in all samples (n=28) were examined. For each flower, the number of petals and sepals was recorded, along with observations of petal morphology and color, and sepal arrangement relative to the rhizome. Measurements included petal length and width, corolla diameter, corolla tube length, and calyx length.

Using a stereomicroscope (Nikon, model SMZ645, serial 1012999) and plant dissection elements (mounted needle, fine-tipped forceps and a scalpel blade), longitudinal cuts were made to the corolla tube to study the internal structures of the flower (stamen filaments and ovary). The number, shape and arrangement of stamens and stigma were recorded. The secretory structures present in the region of the corolla limb (trichomes), described in the genus *Nierembergia* by Cocucci (1991), were also studied. Microscopic observation of the pollen granules was carried out using an optical light microscope (Nikon, model YS100, serial 541327).

### 2.8 Biomass and dry matter analysis of *Nierembergia rivularis*

For each of the block samples collected monthly and described in Section 2.7, the total plant biomass and the biomass of the aerial and belowground parts were measured independently, in order to determine the ratio of the belowground to aerial biomass of *N. rivularis* (Fernández and Johnston, 1986; Melgarejo et al., 2010). Additionally, the proportion of belowground mass relative to the total plant biomass was calculated for each block on both a fresh (green) and dry weight basis. Biomass values are expressed as g per sampled soil block (25 cm^2^ area × 20 cm depth).

Aerial and belowground structures of *N. rivularis* samples were dried separately in a Gallen Kamp Hotbox oven at 60°C until constant weight was achieved. Subsequently dried plants were weighed to determine the dry matter content of each portion (Rosengurtt et al., 1983).

All measurements were conducted using a portable digital scale (SF-400C, 0.01–500 g).

### 2.9 Active compounds identification and quantification

Eight samples of *Nierembergia rivularis* and one sample of *Solanum glaucophyllum* (used as a reference calcinogenic species) were collected from various geographic locations in Uruguay, as detailed in Table 2, each weighing between 150 g and 200 g of fresh plant material. The samples were processed at the Agro-food Laboratory of INIA Las Brujas to tentatively identify and estimate the content of vitamin D3 and its hydroxylated derivatives via Ultra High-Performance Liquid Chromatography with Diode-Array Detection (UHPLC-DAD). The extraction and analysis were performed based on the methodology described by Koshy (1980), with modifications for UHPLC. Compounds were tentatively identified by comparing their UV spectra and retention times to those of certified standards (calcitriol and calcifediol). The total content of hydroxylated vitamin D derivatives was estimated using a calibration curve constructed with calcitriol, and the results are expressed as micrograms of calcitriol equivalents per gram of fresh plant material (μg/g f.p.m.).

**Table 2:** Plant sampling for the quantification of vitamin D and derivatives.

N°	Species	Sample	Date of collection	Department	Geographical reference
1	*Solanum glaucophyllum*	Leaves	Aug. 10, 2022	Montevideo	34°47'30.6"S 56°19'58.0"W
2	*Nierembergia rivularis*	Leaves	Aug. 26, 2022	Tacuarembó	31°56'33.8"S 55°55'53.5"W
3	*Nierembergia rivularis*	Leaves	Aug. 28, 2022	Tacuarembó	32°45'00.5"S 56°25'04.3"W
4	*Nierembergia rivularis*	Leaves	Nov. 1, 2022	Tacuarembó	32°45'00.5"S 56°25'04.3"W
5	*Nierembergia rivularis*	Rhizomes and Roots	Nov. 1, 2022	Tacuarembó	32°45'00.5"S 56°25'04.3"W
6	*Nierembergia rivularis*	Leaves	Nov. 4, 2022	Tacuarembó	32°48'13.5"S 56°30'20.7"W
7	*Nierembergia rivularis*	Flowers	Nov. 4, 2022	Tacuarembó	32°48'13.5"S 56°30'20.7"W
8	*Nierembergia rivularis*	Leaves	Nov. 4, 2022	Tacuarembó	32°48'23.1"S 56°25'17.5"W
9	*Nierembergia rivularis*	Flowers	Nov. 4, 2022	Tacuarembó	32°48'23.1"S 56°25'17.5"W

### 2.10 Statistical analyses

The statistical analyses included the total biomass of *Nierembergia rivularis* and the differences between its aerial and belowground biomass. Initially, the Shapiro–Wilk test was performed to assess data normality. Given the non-parametric distribution of the data, the Mann-Whitney *U* test (α = 0.05) was employed to compare independent biomass components, while the Wilcoxon signed-rank test (α = 0.05) was used to evaluate the paired measurements of belowground mass (fresh vs. dry weight) within the same soil blocks. All statistical analyses were performed using the open-source statistical software R (version 4.3.1).

## 3 Results

### 3.1 Study of growth habits of *Nierembergia rivularis*

*Nierembergia rivularis* exhibits a rhizomatous growth habit, characterized by belowground stems (rhizomes) that may be simple and elongated or form a complex reticulated network. Consequently, to the naked eye, the only visible parts of the plant are its erect leaves and flowers, which emerge directly from the soil. All sites where *N. rivularis* populations were found consisted of lowland natural grasslands prone to flooding or located along the banks of permanent water sources, such as the lake of the *Rincón del Bonete* hydroelectric dam and tributaries of *Río Negro* and *Río Cebollatí* rivers. In all locations, *N. rivularis* grows under direct sunlight, and no populations of this species were observed in the shade of native woodlands.

### 3.2 Characterization of plant assemblages in *Nierembergia rivularis* patches

The six studied sites exhibited heterogeneity concerning the presence of *N. rivularis* and the other plant species present (Table 3). Each sampled 0.5 x 0.5 m quadrant recovered between 5 and 18 total species, with the aboveground cover *N. rivularis* ranging from 1 to 50%. Dry matter and bare soil coverages where typically low, never exceeding 10%. In all cases, plant assemblages were composed exclusively of herbaceous species, usually with a great abundance of grasses (Poaceae), although dominant species varied between sites.

**Table 3:** Plant assemblages in sites with occurrence of *Nierembergia rivularis*. Only species with an aboveground cover of at least 5% in one of the sampled plots from each site are included. Vegetation surveys were carried out in the spring of 2022.

Sampling site A1
Topography: lowland, high humidity. Total species per quadrant: 5 – 15. Dry matter coverage: 5%. Bare soil coverage: 5% – 10%. Main species: ***Nierembergia rivularis* (50%)**. Other species: *Stemodia palustris* A. St.-Hil. (25% – 30%), *Steinchisma hians* (Elliott) Nash (10%), *Cynodon dactylon* (5%).
Sampling site A2
Topography: highland, low humidity. Total species per quadrant: 5 – 15. Dry matter coverage: 5%. Bare soil coverage: 5% – 10%. Main species: *Cynodon dactylon* (80%). Other species: ***Nierembergia rivularis* (5%)**, *Lotus suaveolens* Pers. (5%).
Sampling site B
Topography: mild terrain depression. Total species per quadrant: 8 – 15. Dry matter coverage: 5%. Bare soil coverage: 10%. Main species: ***Nierembergia rivularis* (10% – 40%)**. Other species: *Conyza pampeana* (Parodi) Cabrera (20% – 30%), *Paspalum notatum* (up to 35%), *Steinchisma hians* (0,5% – 15%), *Eleocharis radicans* (Poir.) Kunth (0,5% – 10%), *Cynodon dactylon* (1% – 5%), *Micropsis dasycarpa* (0,5% – 5%).
Location C
Topography: clayey soil characterized by a microtopography of numerous small mounds interspersed with wet depressions. Total species per quadrant: 13 – 18. Dry matter coverage: 10%. Bare soil coverage: 5%.
Sampling site C1
Topography: elevation of the terrain (mound). Dry and rocky area. Main species: *Paspalum notatum* Flüggé (25%) and *Soliva sessilis* Ruiz & Pav. (25%). Other species: *Adesmia bicolor* (Poir.) DC. (10%), *Agalinis communis* (Cham. & Schltdl.) D'Arcy (10%), *Nassella neesiana* (Trin. & Rupr.) Barkworth (5%), ***Nierembergia rivularis* (1%)**.
Sampling site C2
Topography: zone of intermediate elevation and humidity. Main species: *Dichondra sericea* Sw. (50%). Other species: *Paspalum notatum* (30%), *Axonopus fissifolius* (Raddi) Kuhlm. (10%), *Soliva sessilis* (5%), ***Nierembergia rivularis* (1%)**.
Sampling site C3
Topography: terrain depression. High humidity area. Waterlogged. Main species: ***Nierembergia rivularis* (30%)**. Other species: *Cynodon dactylon* (L.) Pers. (25%), *Dichondra sericea* (15%), *Eryngium echinatum* Urb. (10%), *Mentha pulegium* L. (10%), *Steinchisma hians* (5%).

### 3.3 Physicochemical soil properties at *Nierembergia rivularis* sampling sites

Although the objective of the sampling was not to compare the different sites and the results are only descriptive, physicochemical analyses of soil samples indicated that *N. rivularis* can grow in a heterogeneous range of natural grassland terrains (Table 4). Specifically, the edaphic characterization showed variations in texture and chemical composition among the four sampling sites (A, B, C, and D). Soil texture ranged from a sandy type in site A2 (78% sand) to a clayey texture in site D, which presented a clay content of 74%. Sites A1 and B exhibited intermediate sandy-clay properties, with clay contents of 32% and 28%, respectively. Regarding chemical parameters, soil pH varied from acidic in site B (pH 5.5) to slightly alkaline in site A2 (pH 7.8). Concurrently, site C3 presented the highest values for total nitrogen (0.4%), total carbon (4.3%), available phosphorus (27.2 μg P/g), and cation exchange capacity (CICpH7 = 68.5 meq/100g), which was primarily composed of calcium and magnesium. In contrast, phosphorus availability remained below 6.0 μg P/g in sites A1, A2, and B.

**Table 4:** Descriptive physicochemical characteristics of soils at different sites where *Nierembergia rivularis* populations were studied. Because the objective was a descriptive exploratory characterization, a single soil sample was collected from each study site.

	Sampling site			
	A1	A2	B	C3
pH (H_2_O)	6.8	7.8	5.5	6.2
Trit. A. (meq/100g)	2.2	0	3.9	5.1
CICpH_7_ (meq/100g)	25.8	10.2	13.9	68.5
T Bases (meq/100g)	23.6	10.2	10.0	63.4
Sat. Bases (%)	91.5	100.0	72.0	92.6
C. Inorg^α^ (%)	0	0.03	0	0
P citric acid (μg P/g)	3.2	6.0	4.6	27.2
N total (%)	0.1	0.1	0.2	0.4
C total (%)	1.5	0.9	1.8	4.3
Ca (meq/100g)	18.9	8.1	7.0	48.8
Mg (meq/100g)	4.1	1.9	2.4	13.4
K (meq/100g)	0.33	0.17	0.58	0.61
Na (meq/100g)	0.22	0.09	0.09	0.63
Cu (mg/kg)	3.05	1.58	2.61	7.54
Fe (mg/kg)	75.4	52.3	171.6	176.4
Mn (mg/kg)	36.1	34.0	48.9	26.2
Zn (mg/kg)	< 0.2	< 0.2	0.91	2.29
Sand (%)	47	78	53	6
Silt (%)	20	6	18	20
Clay (%)	32	16	28	74
Texture	Sandy-clayed	Sandy	Sandy-clayed	Clayey

Trit. A. = Titratable Acidity; CICpH7 = Cation exchange capacity; T Bases = Total Bases; Sat. Bases = Saturated Bases.

### 3.4 Phenological cycle of *Nierembergia rivularis*

The study revealed that the above-ground structures of *N. rivularis* remained present throughout the year, never disappearing from the soil in winter. This was evident during monthly visits to the sample fields, as well as in laboratory-grown plants under constant light and humidity conditions. Nevertheless, during these visits, fluctuations in the leaf density of *N. rivularis* was observed during dry periods. Notably, significant collective regrowth of the *N. rivularis* population was observed following precipitation events, regardless of the time of year. Flowering began in October and continued until April, from spring to autumn respectively.

### 3.5 Floral dynamics of *Nierembergia rivularis*

#### 3.5.1 Floral cycle

The descriptive observations carried out over the course of the study period show that the floral cycle of *N. rivularis* was characterized by continuous flowering from late October to April, during which the plants repeatedly and continuously produced new flowers throughout the season. Observations at Site C indicated that the flowers remain open at night, with the corolla held in a semi-horizontal position relative to the ground. No nyctinasty movements were observed in the flowers of *N. rivularis*.

#### 3.5.2 Identification of insect species interacting with flowers of *Nierembergia rivularis*

Two insect species were observed interacting with flowers of *N. rivularis*, both during the spring season. The first, observed at Site A on October 2021, around midday, was *Astylus quadrilineatus* (Melyridae). The second one, a small bee from the family Apidae, subfamily Apinae, tribe Tapinotaspidini, was collected at Site B on November 2021, shortly before dusk resting motionless inside a flower of *N. rivularis*.

### 3.6 Morphological studies of *Nierembergia rivularis*

**Fig. 2:**
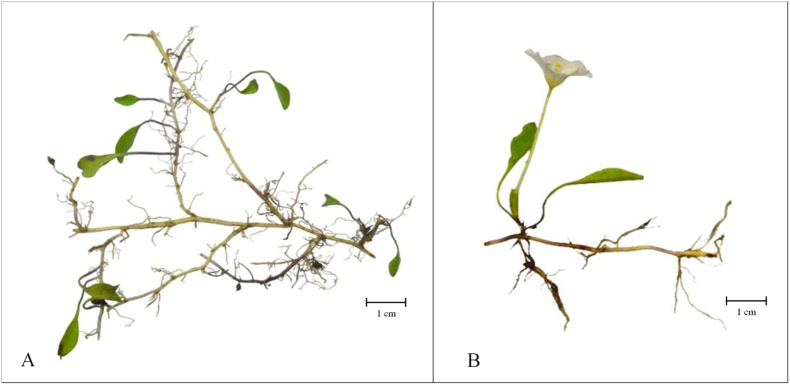
Morphology of *Nierembergia rivularis*. A: Specimen collected during a field visit, showcasing a complex network of subterranean rhizomes. B: The specimen features a simple rhizome with a single floral pedicel and two leaves emerging from a single bud. The elongated, rigid corolla tube positions the hypocrateriform corolla in a semi-parallel alignment.

It was found that *N. rivularis* is characterized by belowground stems in the form of rhizomes, which have a smooth surface and vary in diameter (0.9 to 2.6 mm) and color (purple, white, and brown). The rhizomes may be simple, elongated, and straight, or may exhibit a complex reticulated structure formed by numerous lateral rhizomes (Fig. 2). From the buds present in the rhizomes, one to several leaves emerge alternately, along with floral pedicels and curled adventitious roots, which varied in color from white to brown.

In areas with high competition for belowground space from other plant species, the rhizomes and adventitious roots of *N. rivularis* were found intertwined with the rhizomes and roots of other species, occupying available spaces for growth. This interaction was particularly pronounced with the stout rhizomes of *Paspalum notatum* Flüggé in location C. Consequently, this zone exhibited lower aerial biomass development of *N. rivularis*, characterized by shorter petioles and smaller leaf blades compared to the other sampled areas.

**Fig. 3:**
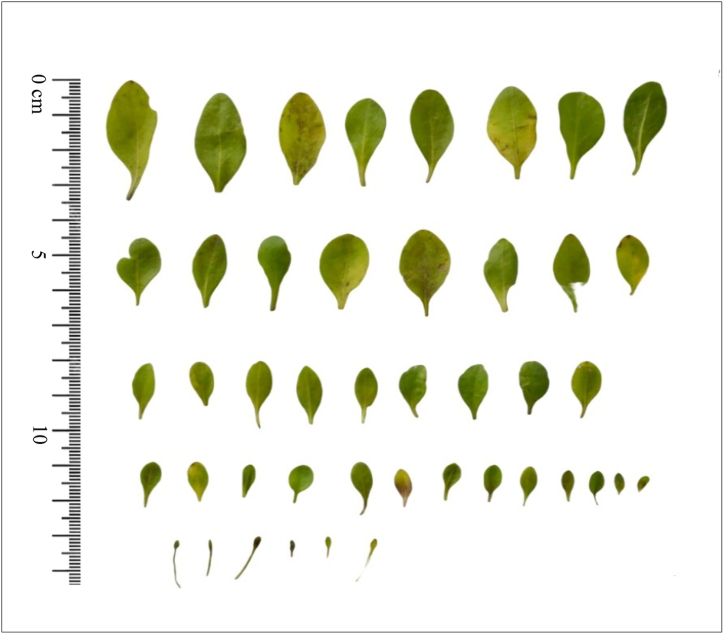
Leaf blades of *Nierembergia rivularis*. The figure illustrates the variation in shape and size of leaf blades across different growth stages of the plant.

The leaves of *N. rivularis* are simple, petiolate, glabrous, and have smooth margins, arranged alternately along the rhizome. In some instances, up to four leaves were observed emerging from what macroscopically appeared to be the same bud (Fig. 2, Fig. 3).

The petioles emerge directly from the soil surface, keeping the leaves upright in a vertical or slightly inclined position. Their lengths vary from 0.3 to 9.4 cm, with longer petioles observed in areas with greater competition for aerial space from other plant species. The petioles exhibit a color gradient, with the portion closest to the rhizome being white, while the distal portions range from violet to green. The leaf blades are spatulate-obovate, with an obtuse apex that tends to be rounded and a tapering base. A reticulated pattern of pinnate venation is evident, and the color of the blades varies from bright green to dark green (Fig. 3).

**Fig. 4:**
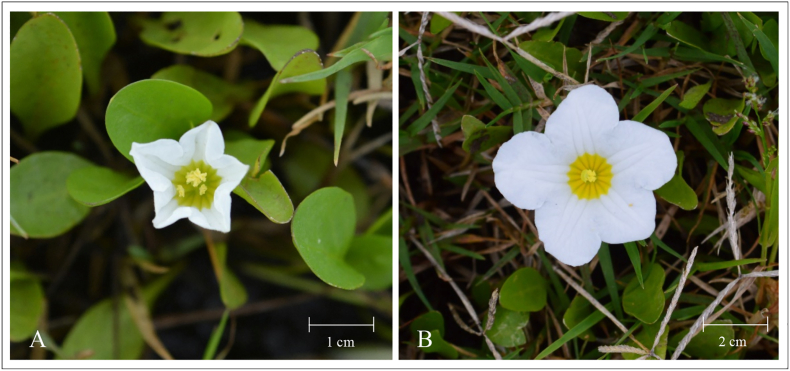
Flower of *Nierembergia rivularis*. The flower comprises five fused petals, with the outer two-thirds exhibiting white coloration and the inner portion displaying yellow. Image A depicts the folding of the semi-open corolla petals, while Image B provides a detailed examination of the coloration and arrangement of the lobes in the fully expanded flower.

**Fig. 5:**
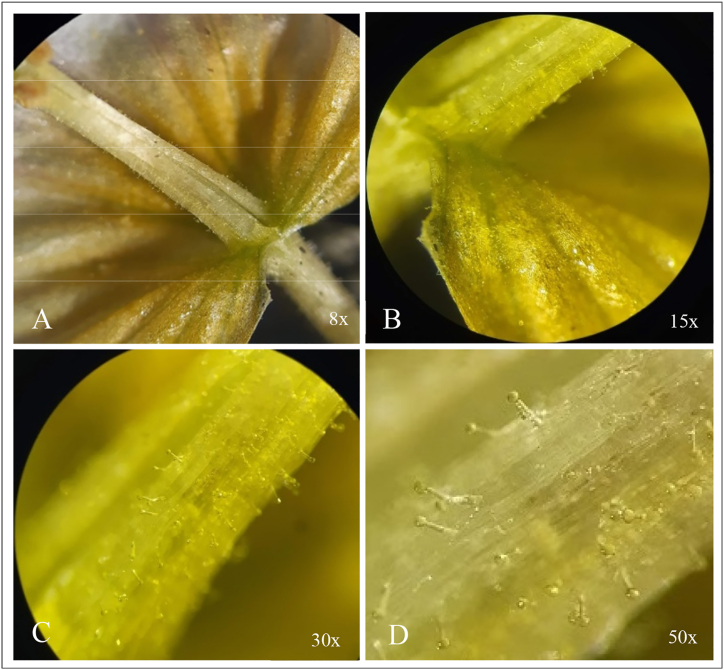
Flower of *Nierembergia rivularis* observed under stereomicroscopy. At lower magnifications (A and B), the extensive distribution of oil-secreting trichomes covering the throat of the corolla and the filaments of the stamens is clearly visible. Higher magnifications (C and D) provide a detailed view of the capitate morphology of the trichomes.

The flowers of *N. rivularis* emerge solitarily from a small pedicel (1 to 2 mm in length) that is inserted into the belowground stem (rhizome). In some instances, one or more leaves were observed emerging from the same bud as the flower. The tubular calyx, composed of five sepals, exhibits a variable length ranging from 10.9 to 16.1 mm. The corolla tube is firm and elongated, measuring between 9 and 58 mm in length, and can be either erect or slightly curved, allowing the limb to position itself semi-parallel to the ground (Fig. 2). The actinomorphic and hypocrateriform corolla invariably consists of five petals that are folded in the closed flower and fused at the base. The outer two-thirds of the limb are white, while the inner third (the throat of the corolla) is yellow, from which the androecium and gynoecium emerge (Fig. 4, Fig. 5). Each petal features a prominent central triangular band of violet on its outer surface, which becomes more pronounced in younger flowers. The data collected on the measurements of the various structures of *N. rivularis* are summarized in Table 5.

**Table 5:** Descriptive morphometric findings on *Nierembergia rivularis* plants in Uruguay. Data summarize monthly observations collected during a 12-month period (March 2021 to February 2022) from two sampling sites (32°44'55.1"S 56°25'17.6"W) and are presented as a descriptive characterization of the species during the study period.

	N	Range (mm)	Mean (mm)	SD
Distance between rhizome nodes	720	2.0 – 34.2	12.6	5.5
Rhizome diameter	720	0.9 – 2.6	1.8	3.2
Leaf blade length	720	50 – 620	170	0.7
Leaf blade width	720	20 – 250	80	0.4
Petiole length	720	30 – 940	240	1.3
Petal length	28	13 – 23	17.1	2.6
Petal width	28	10 – 15	12.4	1.8
Corolla tube length	28	9 – 58	38.3	16.6
Calyx length	28	10.9 – 16.1	13.5	2.3

Stereomicroscopic examination revealed that the ovary is superior, elliptical, and greenish-yellow. Five stamens are fused at the throat of the corolla; three are shorter than the stigma, while two surpass it. The style emerges straight at the corolla throat, curving towards the apex, resulting in a lateral deviation of the stigma. The crescent-shaped stigma is yellowish-green, measuring 1.8 mm in width and 2.0 mm in height, and is inclined towards one side. Nectaries were absent in all examined flowers. Light yellow capitate trichomes cover the petals, pistil, and stamens, predominantly concentrated in the corolla throat (Fig. 5). Microscopic observation confirmed the tetrad arrangement of *N. rivularis*’ pollen grains.

### 3.7 Biomass and dry matter analysis of *Nierembergia rivularis*

The mean total biomass of *N. rivularis* was 25.1 ± 29.4 g of fresh weight (mean ± SD), consisting of 19.6 ± 19.5 g of belowground biomass (rhizomes and adventitious roots) and 8.0 ± 10.4 g of aerial biomass (leaves and flowers) (P = 0.14) (Table 6). Total dry biomass was 6.6 ± 7.7 g, with belowground and aerial components accounting for 6.4 ± 6.2 g and 1.3 ± 1.4 g, respectively (P = 0.008). Total dry matter content was 24.6 ± 7.0%, varying from 29.3 ± 9.4% in belowground structures to 14.0 ± 3.6% in the aerial biomass (Table 6).

**Table 6:** Biomass and dry matter content of *Nierembergia rivularis* samples collected monthly over a 12-month period (March 2021 to February 2022) in two sampling sites (32°44'55.1"S 56°25'17.6"W) in Uruguay. Data are presented as a descriptive characterization of plant biomass during the study period.

	Total plant	BGB (g)^a^	AGB (g)^a^	BGB/AGB Ratio (g)^a^	P	z
**Green Plant Biomass**						
Range (g)	1.8 – 116.2	1.1 – 73.8	0.7 – 42.4	1.2 – 7.1	0.14	−1.0
Mean (g)	25.1	19.6	8.0	2.8		
SD (g)	29.4	19.5	10.4	1.8		
**Dry Plant Biomass**						
Range (g)	0.1 – 27.1	1.6 – 21.4	0.1 – 5.7	2.4 – 15.5	0.008	−2.3
Mean (g)	6.6	6.4	1.3	5.6		
SD (g)	7.7	6.2	1.4	3.8		
**Dry Matter Content**						
Range (%)	12.9 – 41.4	14.9 – 56.1	7.6 – 22.4	---	---	---
Mean (%)	24.6	29.3	14.0	---	---	---
SD (%)	7.0	9.4	3.6	---	---	---

BGB = Belowground Biomass; AGB = Aerial Biomass.

a Biomass found in each sampled soil block of 25 cm^2^ × 20 cm depth.

The proportion of belowground mass relative to total biomass of *N. rivularis* was calculated for each soil block on both a fresh and dry weight basis. Based on fresh weight, the mean proportion of belowground mass was 62.8% (SD = 9.7%), with a range between 54.4% and 87.7% (P < 0.001). On a dry weight basis, the mean proportion was 81.3% (SD = 7.2%), ranging from 70.4% to 93.9% (P < 0.001).

These results show a higher biomass allocation toward belowground structures than toward aerial parts, a distribution pattern that is consistent across both fresh and dry weight measurements. Consequently, the aerial components of *N. rivularis* exhibited a lower dry matter content relative to the subterranean structures.

### 3.8 Active compounds identification and quantification

Hydroxylated derivatives of vitamin D were detected in *Solanum glaucophyllum* and all analyzed samples of *Nierembergia rivularis*. The content of total hydroxylated vitamin D derivatives in the leaves of *S. glaucophyllum* was 52.8 ± 2.1 μg/g f.p.m. (fresh plant material). In *N. rivularis*, the content of vitamin D derivatives ranged from 70.5 ± 1.2 to 200.3 ± 14.4 μg/g f.p.m. across the five leaf samples, and from 83.8 ± 0.07 to 124.3 ± 1.3 μg/g f.p.m. in the two flower samples. The single sample of combined rhizomes and roots presented a content of 176.6 ± 4.6 μg/g f.p.m. (Table 7).

**Table 7:** Vitamin D and its hydroxylated derivatives identified in *Solanumglaucophyllum* and *Nierembergia rivularis* through UHPLC-DAD based on retention times and UV spectra.

N°	Specie	Sample	Total hydroxylated vitamin D derivatives (μg/g f.p.m.)	SD (μg/g f.p.m.)	Vitamin D_3_ (μg/g f.p.m.)
1	*S. glaucophyllum*	Leaves	52.8	2.1	n.d.
2	*N. rivularis*	Leaves	70.5	1.2	n.d.
3	*N. rivularis*	Leaves	90.3	3.2	n.d.
4	*N. rivularis*	Leaves	100.2	1.9	n.d.
5	*N. rivularis*	Rhizomes and roots	176.6	4.6	n.d.
6	*N. rivularis*	Leaves	200.3	14.4	n.d.
7	*N. rivularis*	Flowers	83.8	0.07	n.d.
8	*N. rivularis*	Leaves	116.6	1.7	n.d.
9	*N. rivularis*	Flowers	124.3	1.3	n.d.
	Limit of Detection:		0.13	---	0.01

f.p.m. = fresh plant material; n.d. = not detected.

## 4 Discussion

The results from the surveys conducted at the three farms showed that *Nierembergia rivularis* is highly adapted to natural lowland conditions, including flooded soils and the banks of large watercourses, as documented by Miers (1846). Coincidentally, the assessment of the sampling sites revealed that the highest presence of *N. rivularis* was found in low-lying easily flooded areas (A1, B, and C3), while its occurrence in elevated regions (A2, C1, and C2) was limited. This trend was particularly evident at site C, which exhibited three distinct and adjacent topographic situations (high areas, flat areas and floodplain areas) that were associated with variations in the presence of *N. rivularis*.

The descriptive study of *N. rivularis* conducted in the farms, along with samples maintained under controlled laboratory conditions, confirmed that it is a perennial species with a significant belowground biomass, which enables its long-term persistence in natural habitats. Its flowering period is prolonged (a typical feature of *Nierembergia species*), from spring to autumn season (October to April at 32-33° S in Uruguay). The mischaracterization of its phenological cycle as seasonal, as previously reported in Uruguay (García y Santos et al., 2012) suggesting its disappearance during the winter months, may be attributed to the difficulty of distinguishing these plants from other native grassland species, due to the small size of its leaves and the absence of flowers during that time of year. Additionally, as observed in this study, the development of *N. rivularis* biomass is highly dependent on humidity and precipitation. Low rainfall during autumn and winter may lead to a reduction in the presence of the plant's aerial portions, being the subterranean rhizomes and roots the structures that ensure the permanence of the plants in the fields.

The present study demonstrates the feasibility of maintaining and manipulating *N. rivularis* under experimental conditions outside its natural habitat. The established conditions (20 cm deep soil blocks maintained in plastic containers with proper drainage, periodic irrigation and direct sunlight exposure) were suitable for observing the plant outside its environment, allowing to use this technique in future experimental trials. In addition, physicochemical analyses revealed that *N. rivularis* can grow in a heterogeneous variety of soils, including sandy, clayey and mixed-texture soils, with no major fertility constraints in terms of pH (which ranged from slightly acidic to slightly alkaline), cation exchange capacity, or base saturation (Rabuffetti, 2017).

At Site C3, the observed patterns suggest that soil physical properties and underground space competition might exert a strong influence on *N. rivularis* populations. Despite possessing the highest water retention capacity due to elevated clay content, alongside optimal nutrient availability (maximal P, Ca, Mg concentrations and cation exchange capacity), the highly compacted nature of the soil combined with the dense rhizomatous network of *Paspalum notatum* could have limited the expansion of *N. rivularis* underground biomass. This physical and competitive restriction may have ultimately prevented the species from reaching its maximum vegetative potential, leading to a concomitant reduction in aerial biomass. Consequently, these findings propose a plausible ecological mechanism for developing targeted management strategies.

This study represents the first morphometric investigation of *N. rivularis*, providing a detailed description of its morphology throughout the entire phenological cycle and contributing to the existing literature on the subject. Key characteristics that distinguish this species include its rooting stems that form subterranean rhizomes, glabrous spatulate leaves emerging directly from the soil surface, and the morphology of its flowers (long, firm corolla tube and hypocrateriform corolla positioned semi-parallel to the ground, exposing its reproductive structures). Beyond these macro-morphological traits, the tetrad pollen arrangement is a distinctive feature of *N. rivularis*, as confirmed in the plants studied. This palynological evidence allows for clear differentiation from other similar rhizomatous species, such as *N. repens* (single pollen grains), with which it was incorrectly synonymized in past taxonomic revisions (Di Fulvio, 1976; Millán, 1941; Cocucci and Hunziker, 1995; Tate et al., 2009).

A characteristic purple coloration is evident in the rhizomes, leaf petioles, and triangular segments of the flower petals. This pigmentation, which is most intense in young plants, has been attributed to anthocyanin production in these structures and likely serves to attract pollinating insects to the plant's reproductive parts (Tate et al., 2009).

Microscopic observation of the flower structures allowed the description of the shape, coloration, and distribution of glandular trichomes (elaiophores) as noted by Cocucci (1991) in other *Nierembergia species*. Additionally, a specimen of a native bee from the tribe Tapinotaspidini was found on a flower of *N. rivularis*. The relationship between oil-collecting bees and oil-secreting flowers has been documented in Argentina (Cocucci, 1991); however, this interaction has not been previously reported in Uruguay, where limited information is available regarding native bee populations (Santos S., pers. comm., 2023). In nature, few botanical species possess nectarless oil-secreting flowers. *Nierembergia* is the only genus within the Solanaceae family exhibiting this floral type (Cocucci, 1991; Maubecin et al., 2021). While self-pollination has been ruled out for *N. rivularis* (Cocucci, 1991), its primary mode of reproduction seems to be asexual (clonal), with rhizome growth serving as the main means of propagation in the field (Cocucci, A.A., pers. comm., 2022). The absence of nectary and the persistence of oil-secreting structures (elaiophores) in these species throughout their evolution has contributed towards the preservation of oil-collecting bee populations, which are involved in the pollination of multiple botanical species beyond *Nierembergia*, playing a significant role in native natural field ecosystems (Cocucci, 1991; Tate et al., 2009; Maubecin et al., 2021). Although interactions between insects of the *Astylus* genus and *Nierembergia* flowers have been reported previously, the effectiveness of these insects as pollinators has been dismissed (Cocucci, 1991). Field observations during the night revealed that the flowers of *N. rivularis* remain open overnight, a characteristic not previously documented in the literature. This enables them to serve as a refuge for solitary, oil-collecting bee species, suggesting potential interactions with nocturnal insects. However, the ecological importance of these insects should not be overestimated, as pollination efficiency and reproductive success have not been experimentally evaluated.

The available literature, along with the descriptive findings on the life cycle and morphology of *N. rivularis* presented in this study, provides a foundation for formulating hypotheses regarding the seasonal occurrence of enzootic calcinosis outbreaks, characterized by clinical manifestations and mortality from spring (September) until autumn (April) (García y Santos et al., 2012; Machado et al., 2020a,b). The confirmed perennial nature of *N. rivularis* rules out the hypothesis attributing the disease's seasonality to the plant's disappearance during autumn and winter (García y Santos et al., 2012). The seasonality is likely linked to the interaction of two primary factors. First, the presence of *N. rivularis* in the field diminishes (with smaller leaves) compared to winter species in natural grasslands, which exert greater competitive pressure. Second, seasonal climatic variations (characterized by lower temperatures, reduced precipitation, diminished sunlight exposure, and decreased UV radiation intensity), likely contribute to the reduction of calcinogenic metabolite synthesis by the plants during autumn and winter (Mello and Habermehl, 1991; Boland et al., 2003). These environmental conditions may induce a state of metabolic dormancy in the plants, leading to a decline in processes related to vegetative growth and rhizogenesis, which is regulated by vitamin D and its sterols (Mello and Habermehl, 1991; Boland et al., 2003). Further studies are necessary to validate these hypotheses, particularly those focusing on the metabolism of vitamin D in calcinogenic plants.

According to official metrics (INUMET, 2026), the study period (March 2021–February 2022) was marked by a severe multi-seasonal drought, evolving through a deficit-ridden autumn (–18.8% rainfall anomaly), a dry winter interspersed with localized June rains (+75.3%) and severe July frosts, a spring defined by a wet September (+50.0%) followed by a dry October (–66.4%), and a summer characterized by the most deficit-ridden December since 1980 (15.7 mm) alongside a historic record-breaking heatwave in January (44.0 °C). These severe meteorological anomalies recorded during the study period may have exerted strong functional pressures on the architecture and biochemical profile of *N. rivularis*, offering a plausible framework to interpret the collected data. Clonal herbaceous weeds inhabiting natural grasslands frequently tend to adapt to prolonged water and thermal stress by reducing open vegetative elongation and accelerating aerial senescence (Lemaire et al., 2000; de Kroon et al., 2012). To prevent potential structural dehydration and sustain hydraulic connectivity under temperatures that peaked during the historic 44.0 °C heatwave in January, the plant might have prioritized belowground biomass allocation. This evolutionary defense mechanism could explain the high structural persistence and dense fresh and dry weights tracked monthly within our standardized 25 cm^2^ × 20 cm sampled soil blocks; the subterranean rhizomatous network may have acted as a carbohydrate sink, potentially guaranteeing population survival despite consecutive dry seasons and winter frosts. Conversely, the sudden, intense winter and summer precipitation pulses appear consistent with a rapid, opportunistic rehydration of these underground reserves, which likely allowed the plant to maintain its vegetative persistence without relying on expansive canopy growth. Furthermore, these fluctuating climate extremes might have modulated the secondary metabolism and toxicological risk profile of the species. Under restricted growth conditions induced by severe drought, the rate of calcinogenic compound synthesis could potentially outpace cellular elongation (Herms and Mattson, 1992; Selmar and Kleinwächter, 2013). This stress-induced accumulation effect may concentrate active hydroxylated vitamin D-metabolites within the remaining vegetative and reproductive structures. Consequently, the high organ-specific toxicity detected by HPLC across the warm sampling seasons is consistent with historical observations where drought episodes in Uruguay appear to have modified natural pasture composition, potentially favoring the abundance and metabolic concentration of toxic *Nierembergia* lineages over dominant forage grasses (Mederos et al., 1991).

In this context, the results of this study showed that the biomass of *N. rivularis* is distributed heterogeneously. In green plants, the belowground structures (rhizomes and adventitious roots) exhibit a mass up to seven times greater than that of the aerial structures (leaves and flowers). These differences were even more pronounced in dry weight measurements, with belowground biomass being up to 15 times greater than aerial biomass.

The rhizomatous system of *N. rivularis* is fundamental for its persistence and vegetative propagation. Its rooting stems (rhizomes) likely reduce the productivity of natural grasslands by competing for belowground space, nutrients, and water with co-occurring species, a phenomenon previously described in other rhizomatous taxa such as *Cynodon dactylon* (L.) Pers. (Ríos, 1999; Font Quer, 2000). These characteristics are essential when formulating control plans for *N. rivularis*. Machado et al. (2020b) recommended a chemical treatment involving a combination of three systemic herbicides (2,4-D, Metsulfurón, and Tordon) along with strategic grazing management in affected areas. However, significant considerations regarding the feasibility of chemical control must be taken into account, not only because *N. rivularis* plays a critical role in the preservation of native oil-collecting bees as discussed before, but also because it grows near water sources and flood-prone areas. In Uruguay for example, these measures are currently limited by legal restrictions, which establish minimum exclusion distances from natural watercourses and surface water sources for both aerial and mechanized ground applications of agrochemicals, due to potential environmental consequences (DGSA Resolution No. 129/008 - MGAP).

As an alternative to chemical control, the competitive establishment of rhizomatous species such as *Paspalum notatum* could be suggested, which has been studied in Uruguay as a tool for improving natural pastures (Giorello et al., 2021). This is a perennial, summer-growing native grass of Uruguay, characterized by their versatility and adaptability to various soils, high potential for producing nutrient-rich forage, and ability to restore degraded pastures while reducing weed growth (Giorello et al., 2021). However, this recommendation remains hypothetical, as no experimental evidence in the control of *N. rivularis* has been presented.

The findings of this research confirm that *N. rivularis* accumulates hydroxylated derivatives of vitamin D in both its aerial (leaves and flowers) and belowground structures (rhizomes and roots). These results are similar to those reported in *S. glaucophyllum*, which exhibited vitamin D sterols across all plant parts (Curino et al., 2001). Although the precise molecular composition of the compounds could not be identified, the co-elution of the peaks observed in UHPLC to the standards for calcitriol and calcifediol suggests that they are chemically similar. This identification remains tentative because Diode-Array Detection (DAD) relies solely on UV spectra and retention times, which cannot resolve specific molecular structures. Molecular identification of the vitamin D derivatives in these plants will require more specific and sensitive techniques, such as liquid chromatography coupled with mass spectrometry (Jäpelt and Jakobsen, 2013). *Solanum glaucophyllum,* the most extensively studied calcinogenic plant in the literature, consistently presented high levels of hydroxylated vitamin D derivatives across various quantitative studies (Boland et al., 2003; Jäpelt and Jakobsen, 2013). Research assessing the toxicity of four plants categorized *S. glaucophyllum* (cited as *S. malacoxylon* Sendtn.) and *Cestrum diurnum* as having greater calcinogenic activity than *Trisetum flavescens* and *N. riograndensis* (cited as *N. veitchii* Hook.) (Mello and Habermehl, 1995). The effects on the calcemia and phosphatemia of poisoned animals were attributed to the presence of compounds similar to calcitriol, both in free (lipophilic) and glucoside-conjugated (hydrophilic) forms (Mello and Habermehl, 1995).

In this study, we analyzed leaf samples of *S. glaucophyllum* using HPLC to establish a reference value for comparing hydroxylated vitamin D derivatives in *N. rivularis*. The results obtained for *N. rivularis* were notably greater than those found in *S. glaucophyllum* samples. Further studies are needed to draw conclusions regarding the toxicity of *N. rivularis*, including the identification of the molecular forms of hydroxylated and glycosylated vitamin D derivatives, and performing in vivo experiments with its extracts, similar to those previously conducted with other calcinogenic species (Peterlik et al., 1976; Wasserman et al., 1976; Peterlik and Wasserman, 1978; Riet-Correa et al., 1987; Prema and Raghuramulu, 1994; Mello and Habermehl, 1995; Boland et al., 2003). Nevertheless, the toxicity and calcinogenic effects of *N. rivularis* are well documented in sheep and cattle (García y Santos et al., 2012; Machado et al., 2020b; Schild et al., 2021). The results of this study suggest that *N. rivularis* has a high capacity to metabolize and store hydroxylated vitamin D derivatives, a characteristic that is distinctive of the toxic effects caused by solanaceous calcinogenic plants (Boland et al., 2003; Mello, 2003; Jäpelt and Jakobsen, 2013).

Research on the content of vitamin D derivatives in calcinogenic plants is limited and often plagued by methodological discrepancies that make it difficult to compare results (Jäpelt and Jakobsen, 2013). Developing standardized procedures for sampling, extraction and advanced analysis techniques (HPLC/MS) would facilitate comparative studies of vitamin D derivative content among different calcinogenic species in Uruguay and other South American countries, as well as investigations into the factors that determine variability in the toxicity of these plants.

## 5 Conclusions

This descriptive study confirmed that *N. rivularis* is a perennial species characterized by significant belowground biomass, which enables its long-term persistence in natural habitats. The main requirements for its growth are related to high soil humidity (preferring flooded surfaces or margins of water sources), direct sunlight exposure and mixed soil texture which, together with low competition from rhizomatous species, allows the optimal development of its belowground biomass.

The results show that *N. rivularis* accumulates hydroxylated metabolites of vitamin D in all plant structures, which explains its calcinogenic properties, closely associated with toxic effects in several animal species. However, the analytical approach remains preliminary because the compounds were only tentatively identified through UHPLC-DAD based on retention times and UV spectra. Molecular confirmation through LC-MS/MS or related analytical techniques would be necessary to definitively identify the metabolites involved.

## CRediT authorship contribution statement

**Marcela Preliasco:** Conceptualization, Data curation, Investigation, Visualization, Writing – original draft. **Ary Mailhos:** Conceptualization, Investigation, Writing – review & editing. **Facundo Ibáñez:** Data curation, Formal analysis, Investigation, Validation, Writing – review & editing. **Alejo Menchaca:** Conceptualization, Funding acquisition, Resources, Supervision, Writing – review & editing. **Aldana Balserini:** Investigation, Resources, Writing – review & editing. **Ana Celina González:** Investigation, Writing – review & editing. **Franklin Riet-Correa:** Conceptualization, Investigation, Writing – review & editing. **Rodolfo Rivero:** Conceptualization, Funding acquisition, Resources, Supervision, Writing – review & editing.

## Declaration of competing interest

The authors declare that they have no known competing financial interests or personal relationships that could have appeared to influence the work reported in this paper.

## Data Availability

Data will be made available on request.
